# Lived Experiences of Adults with Sickle Cell Disease: A Qualitative Study, Dar es Salaam, Tanzania

**DOI:** 10.24248/eahrj.v6i2.699

**Published:** 2022-11-30

**Authors:** Dickson Ally Mkoka, Rehema Nkingi

**Affiliations:** a Department of Clinical Nursing, School of Nursing, Muhimbili University of Health and Allied Sciences (MUHAS), Dar-es-Salaam, Tanzania; b Critical Care Nurse Specialist, Muhimbili National Hospital, Dar-es-Salaam, Tanzania

## Abstract

**Background:**

Sickle Cell Disease (SCD) is most common genetic disorder and its prevalence in sub-Saharan Africa is increasing. Despite increased survival rates, experiences of adults living with SCD in Tanzania is not well explored. This article provides perceived causes of pain crisis, pain self-management approaches and psychosocial implication of SCD.

**Aim:**

This study aimed at exploring experiences of adults living with SCD regarding pain triggering or aggravating factors; self-management for pain; psychosocial-economical implication of SCD and coping mechanism used by individuals living with SCD

**Methods:**

A qualitative study design was chosen using in-depth interviews with adults living with SCD to explore their experience of living with SCD. Fifteen adults aged 18 years and above living with SCD were interviewed. Data were analyzed by using content analysis approach.

**Findings:**

Four categories emerged that described experiences of individuals with SCD. The four categories are; “Pain Triggering and Aggravating Factors” describing participants' perceived factors causing pain in SCD; “Self-care remedies for the pain” referring to participants' methods for self-management of pain; “Psychosocial-economic impact of illness” referring to participants' experience of implication of illness on social and economic life and “Dealing and coping with illness” referring to experience of participants on management and coping strategies used to live with the illness.

**Conclusion:**

Individuals with SCD experiences several episodes of pain that affect their quality of life. Pain episode can be triggered or aggravated by various factors. Several approaches are used by individuals with SCD to self-manage the pain including taking rest, drinking plenty of water or using pain relieving medication. Care for individuals with SCD should be comprehensive and include proper management of pain, health education on home-based intervention for sickle cell pain, supportive services to deal with psychosocial implications of SCD and improving coping strategies to live with the illness.

## BACKGROUND

Sickle Cell Disease (SCD) is a potentially overwhelming genetic disorder accompanied by episodes of painful attacks that affect quality of patient's life. The disease is due to genetic defect of hemoglobin, a molecule in red blood cells that carries and delivers oxygen to cells throughout the body. People with this disease have abnormal hemoglobin known as Hemoglobin S (HbS) which alter the shape of red blood cells to sickle shaped or crescent. SCD is prevalent in many countries including Africa and is the most common genetic disorder.^1–4^ It is estimated that 16% of the population in Africa has a sickle hemoglobinopathy which is the highest proportion worldwide.^4^ The symptoms for the disease usually begin in early childhood and most present with low number of red blood cells, repeated infections and periodic episodes of pain.

Despite high mortality during childhood, several others survive to adulthood. However, living with the SCD has been a challenge to many adults with the disease. The effects of SCD are multi-dimensional, ranging from causing high morbidity, and reducing the quality of life, to imposing a high socio-economic burden on individuals, and families.^5–7^

Pain is one of the major problems of SCD. SCD pain occurs when red blood cells with the abnormal form of hemoglobin become sickling (deforming), preventing blood flow, and thus producing ischemia, hypoxia, and possible tissue damage. Pain is gradual in SCD, affecting all aspects of life.^8–12^ Management of SCD pain has to be holistic (take into account all aspects of patient's life) without forgetting mental and social factors and not just the physical symptoms of the disease, but this has not been the case among health care providers in hospitals.

Poor painful crises management can lead to increase in frequencies of crises, and later to chronic pain that may result in recurrent hospital admissions, frustration, and loss of precious time for adult's daily activities. This can have direct consequences on economy and implicate social life. Little is known about experience of adults living with SCD in Tanzania. This study is therefore set to explore the experience of adults living with SCD and in particular, how they perceive factors triggering SCD related pain episodes, how they self-manage such pain; their understanding of psychosocial-economic implications of SCD and their coping mechanisms that help them to live with the illness. Such understanding is useful in developing comprehensive intervention including preparation of educative sessions on self-care management, prevention or reduction of crisis occurrences programs and counseling targeting reduction of psychosocial impact of the disease as well as improving their coping mechanism.

## METHODS

### Study Design and Setting

A qualitative study design was used. The study conducted in-depth interview of adults living with SCD to explore their perceptions regarding causes of the pain they experience, how they manage pain, psychosocial and economic effects of the illness.

This study was done at Muhimbili National Hospital (MNH), in Dar-es-Salaam, Tanzania. The hospital is a tertiary, referral and teaching hospital that serves the whole country. It is the largest hospital with 1,500 bed facility, admitting 1,000 to 1,200 inpatients per week. The hospital has 2700 employees of whom 300 are doctors and specialists, while 900 are registered and enrolled nurses. It has a General Haematology Clinic that enrolled over 6000 individuals with SCD since 2006.

### Study Participants

This study included participants who have been living with SCD for more than 18 years, confirmed to have SCD screened at the Hematology Unit and consented to be enrolled in the study.

### Sample Size and Sampling Technique

A total of fifteen (15) participants were interviewed in this study. The sample size was not predetermined. However, the researchers stopped with 15^th^ interview after noting repetition of information with little or no new insight in relation to our research questions. Selection of participants was purposely done to include those who has been living with SCD for more than 18 years and attending the General Hematology Clinic.

### Data Collection

Interviews were conducted between March and May 2017. The authors in collaboration with nurses at the hematology unit identified participants who met inclusion criteria and requested to participate in the study. All interviews were conducted in a convenient room to avoid interruption during interview sessions. Interviews were conducted in Kiswahili language by RCN, who is a nurse, a Kiswahili native speaker with prolonged experience of working in Haematology Unit at the Hospital. Interviews were conducted using semi-structured interview guide with open-ended questions on issues regarding causesof pain; self-management of pain, psychosocial and economic effects of the illness. However, the interviewer remained open to other new emerging issues with regard to participants' experience of living with SCD. The interviews were recorded using digital recorder with permission from study participants to ensure that all information was captured. Field notes were also taken and preliminary analysis of data was done following initial data collection. This enabled the researchers to gain an insight of emerging issues which were followed up in subsequent interviews.

### Ethical Consideration

Ethical approval to conduct this study was obtained from the Senate Research and Publications Committee, which is the Institutional Review Board of Muhimbili University of Health and Allied Sciences. The permission to conduct the study was given by MNH. Before interview, researchers obtained written informed consent from the participants.

### Data Analysis

To ensure accuracy and completeness of data, interviews were reviewed daily. Recorded interviews were transcribed verbatim and then translated into English. Translation was done in by two authors (DAM & RCN) who use Kiswahili language as their mother tongue. Qualitative content analysis as described by Graneheim and Lundman^13^ was employed in analyzing the data. Transcripts were then read and re-read by all authors to familiarize with the data and generate insight on the contents. Each transcript was then analyzed for identification of text (meaning units) related to causes of pain, self-management of pain at home, perceived effect of the illness in social and economic life and the management and coping methods developed to live with the illness. The meaning units were condensed and codes were then extracted. Similar codes were sorted to form categories reflecting the manifest content of the text and similar categories were organised into themes reflecting the latent content of the text. Data from field notes were used as supportive information in clarifying concepts that emerged during analysis of the transcripts.

## RESULTS

The age of participants ranged from 18 to 45 years. Nine participants were females and 6 were males. All participants were not married except 6 females who were divorced. Seven participants had primary school education, four secondary school education and 4 had college education. Only 2 participants were employed and the rest were not employed. Only 4 out of the 15 participants owned health insurance treatment cards.

### Lived Experience of Individuals with SCD

During analysis of the interviews, four categories emerged that described the experiences of participants living with SCD. Categories and selected codes are presented in Table 2.

**TABLE 1:** Demographic Information of Participants (N=15)

Demographic characteristics	Numbers (%)
Age range (Years)	
18-20	2 (13.3%)
21-30	8 (53.3%)
31-40	4 (26.7%)
41-50	1 (6.7%)
Gender	
Male	7
Female	8
Marital status	
Married	0
Single	13
Divorced	2
Occupation	
Employed	0
Self-employed	13
Student	2
Pain experienced in the past six months	
No pain	1
Moderate pain	4
Severe pain	10
Possession of National Health Insurance Fund (NHIF) card	
Have NHIF card	3
Have no HHIF card	12

**TABLE 2:** Categories and Selected Codes Describing Experiences of Adults Living with SCD

Experience of Adults with SCD	Selected Codes	Category
What causes and exacerbates pain	-Pain varies with frequency, intensity and severity -Pain can start by itself -Getting wet and cold from rain -Stressing events -Having infected wounds -Energy demanding work -Decreased water intake	Pain triggering and aggravating factors
How to deal with pain at home	-Rest by sitting or sleeping -Distracting pain with other activities -Massaging using hot water -Self-medicating with pain killers -Drinking plenty of water	Self-care remedies for pain
Psychosocial and economic effect of illness	-Poor school attendance due to frequent hospital admission -School dropout caused by chronic illness -Lost job due to chronic illness -Stigmatized once disclosing the illness -Difficulty in getting life partner -Altered family process due to frequent admission -Divorced due to illness induced infertility	Psychosocial-economic implication of illness
How to manage and live with illness	-Seeking hospital care if pain persist -Attending hospital for health checkup -Knowing and receiving right treatment -Seeking healing from tradition healers -Praying daily for divine healing -Hoping and believing in God for healing	Managing and coping with illness

### Pain Triggering and Aggravating Factors

Pain was mentioned as a common symptom experienced by participants in the course of their illness. The frequency and severity of pain experienced varied among participants. It was noted that some participants experience severe pain daily while others experience pain intermittently and that the pain can either be moderate or severe.

Participants in this study, shared their experiences on what they view as factors that trigger or aggravate pain. Although some participants reported to experience pain spontaneous without any obvious cause, several others reported to experience severe pain after being wet from rain or during cold weather. Some participants reported to experience pain whenever they go through stressful situations. It was noted that having an infected wound could trigger pain among patients living with SCD. One participant shared on how she started to experience severe pain after having an infected ulcer in one of her legs.

Some participants reported that when they drink little amount of water and became dehydrated, they end up getting severe pain. Others mentioned that involvement in energy demanding work like strenuous physical exercise or other activities that demand more energy was mentioned to trigger severe pain as attested by one of the participants.

*“I feel severe pain when I do excessive exercise. Another thing is that I get pain when I drink little water or if I don't drink water at all.”**(Participant 15)***

### Self-care Remedies for Pain

Participants reported various modalities they use to alleviate pain or get relief from pain. Some reported that when they get pain, they sleep or sit without doing anything and the pain disappear or get reduced. Some reported that drinking plenty of water or fluids helped to alleviate pain crisis once it occurs. Though participants are normally advised to drink about 3 liters per day, some participants reported to have been drinking more or less than what is recommended. However, other participants reported to get difficulty to achieve the required amount of water per day and mentioned that, achieving that goal need some extra effort as mentioned by one participant below.

*“I drink that amount because I don't like water. I try my best to drink but I cannot finish two liters in a day. I don't like water like others do, so, drinking that amount needs some effort”. **(Participant 6)***

Wearing warm clothes during cold weather was also mentioned as one of the approaches that helps to alleviate pain or prevent painful crisis. Others preferred to be massaged or take bath with hot water to alleviate pain. However, some participants tried to distract pains by continuing with their normal activities.

All participants reported self-treatment by using analgesic tablets as a way of alleviating pain. However, it was noted from the interview that, the medication taken are not necessarily prescribed and that amount of drugs taken depends on their effectiveness in relieving the pain, exposing them to drug toxicity.

### Psychosocial-Economic Implicationof Illness

Almost all participants reported to have missed classes and examinations in schools due to frequent sickness from SCD. Some participants dropped out of school completely due to sickness.

Other participants shared their concerns about financial status, some mentioned that they lost their jobs because of SCD illnesses. Few reported to have been fired from work by their employers due to frequent sickness. Other participants reported to have been stigmatized in their working place after disclosing their disease status. One participant narrated on how he was terminated from work after his employer discovered that he was suffering from SCD.

Participants also shared the challenges they are experiencing when want to establish family life. Some confirmed to have been in relationship or marriage that ended into breakdown or divorce. One female participant described how her fiancée broke the relationship after she disclosed her SCD status. Other participants expressed that, due to frequent illnesses and sickle cell crises, or miscarriages, their in-laws become intolerant and convinced their sons to divorce. A participant who was divorced due to frequent sickness and miscarriages stated as follows:

*“Yes, my husband knew, but after the marriage the problems started again, I was attending hospital frequently, sometimes admitted, sometimes this, till when I had my first child. After the delivery, the frequency of sickness declined, but he knew this would happen again and so I was divorced and went back home”. **(Participant 7)***

### Managing and Coping with Illness

Participants in this study shared different approaches they use to self-manage pain. However, once pain become severe or persistent almost all participants reported going to hospital for further management. Several participants mentioned that once in hospital, they were treated by injectable Diclofenac or Tramadol and IV fluids. It was noted from the interviews that, most of the participants are known by health care providers and often given right management.

Some participants were concerned of the high cost for treatment. Others narrated how some hospitals denied treatment and referred them to MNH for only pain management. Lack of patients' involvement in care was another concern as narrated by one participant below:

*“I once went to the hospital and I had very severe pain! The doctor forced me to take tablets only saying that it would help while I knew that the tablets at that time would not help me at all”. **(Participant 11)***

Some participants confirmed attendance to traditional healers, taken by their relatives after associating the disease with witchcraft. Other participants acknowledged to practice religious practices such as prayers and considered it to be helpful in promoting hope and inner strength to face the challenges emanating from living with SCD. Some confirmed to pray whenever they are in pain or very sick and had faith that one day they will experience divine healing from God.

*“We pray every day. I pray daily for a relief from the disease. I pray daily, I believe, I believe in God and He will heal me”. **(Participants 1)***

## DISCUSSION

This study explored the experience of adults living with SCD. The study found several factors that trigger or aggravate pain crisis and approaches that individuals living with SCD use to self-manage the pain at home. Furthermore, the study found several psychosocial and economic implications resulting from living with SCD and various strategies used by such individuals to cope with the illness.

### Pain Triggering or Aggravating Factorsand Self-care Remedies for Sickle cell related Pain

Individuals with SCD experience episodes of pain that can be very severe and result in multidimensional problems that affect their quality of life. However, the study found variation on how individuals with SCD experience such pain. Most participants in this study reported to experience intense and continuous pain, however, for others, the pain just occurred occasionally and moderately. This variation could better be described by genomic variability, whose exploration can be used for identification of susceptibility to chronic pain experienced by individuals living with SCD.^9,13–20^

Several studies have demonstrated how genomic information is crucial determinant of chronic pain and hence recommend the need for evaluation of genomic variables for predicting pain chronicity in individuals with SCD.^9,17^

This study found several factors that trigger or aggravate pain in SCD. Cold weather or becoming wet was found to be the main triggering factor for painful episodes in individuals with SCD. The study in Ghana by Tewari and his colleagues^9,17,21^ showed that extreme cold weather coincided with the rainy season precipitate severe pains in patients with SCD. However, it was noted in this study that, in most cases, patients with SCD were not aware of this and recommend routine advice to avoid getting cold or being wet.^21^

It has been reported in other studies that, hospital admission with sickle pain increases in cold winter months even when episodes with overt infection were excluded, and speculated that this may be due to increased blood viscosity and cold diuresis.^20,22,23^ Also, dehydration, infection and other life stressors were found to either trigger or aggravate pain in individuals with SCD in this study. Apart from this, energy-demanding activities such as running exercise were revealed in this study to be among pain aggravating factors. Several other studies demonstrated the pathophysiological link between physiological or emotional stress and the occurrence of vaso-occlusive crisis among patients with SCD.^19,23–27^ It is now known that mental stress decreases microvascular blood flow, which may trigger episodes of vaso-occlusive crisis among patients with SCD.^25^ Also dehydration in SCD, makes blood more viscous and hence increasing risk for vaso-occlusive crisis leading to severe pain.^27,28^

The study revealed several approaches by which individuals with SCD use to deal with sickle cell pain. Most of these approaches are non-pharmacological including resting once pain arises, massaging using hot water and drinking plenty of water. Also, distraction wasalso found to be an effective intervention that some patient use to relieve themselves from pain. Non-pharmacological approaches are considered to be preferred methods for pain relief among individuals with SCD.^29–31^ Other non-pharmacological pain relief approaches that are used elsewhere by SCD patients are acupuncture, aromatherapy, relaxation, massage, music, vibration, therapeutic exercises and self-hypnosis.^30,31^

Exploring the usefulness of these approaches and their effectiveness in relieving pain among patients with SCD is recommended. Introduction of these approaches could widen patients' choice for non-pharmacological approaches for pain relief which are considered to have fewer side effects.^30,31^ However, similar to other studies^32–34^ self-medication with non-steroid inflammatory drugs such as diclofenac, acetylsalicylic acid (aspirin), and acetaminophen was found in this study to be common among patient living with SCD. While this could risk users with irrational use of these drugs, the study recommends thorough assessment and individualization of therapy coupled with the use of non-pharmacologic and pharmacologic approaches.

### Psychosocial-economic Implication of SCD and Coping Mechanism Utilised by SCD Patients

Findings in this study reveal that, individuals with SCD encounter psychological, sociological and economic challenges affecting their quality of life. Frequent illnesses and admissions to hospitals was found to be major cause of poor academic achievements for some participants in this study. Frequent school absence in children with SCD was found to be a predictor of poor academic performance in other countries.^35–37^ This poor academic performance has implication later in adult life, as most of them end up with low level of education hence getting difficulties in securing well-paid jobs.

Those who managed to advance in school still faced difficulties in securing stable employment due to stigmatisation or being terminated from their jobs due to frequent illness. Furthermore, physical disabilities, frequent acute and chronic pain episodes or other complications are major reasons leading to SCD patients being fired from work and leaving them economically unstable.^12,38,39^ Additionally, this study shows that, individuals living with SCD fail to establish strong courtship, experience difficulties in getting married and establish stable family life. Some female participants shared how they went through unstable relationship with their sexual partners; experienced break up of relationship and living single life after being divorced because of frequent illness or miscarriage. Other findings show how SCD individuals experience marital dysfunctions or difficulties with interpersonal relationship with sexual life partners.^12,40^ Hospital facilities should include genetic counselling and other social services targeting provision of psychosocial support for adults with SCD.

Despite the challenges implicated by the diseases, this study found a number of adoptive coping mechanisms utilised by individuals with SCD to live with the illness. The study revealed the use of religious practice such as prayers as useful means utilised by some patients as a source of hope and inner strength to cope with the disease. A study done in Nigeria found that people living with SCD commonly used religious practices as an affective coping strategy.^38^ Religious practices such as prayer meetings are considered as effective supportive mechanism for individuals with SCD and their close families to cope with the illness.^41–44^ Religious and spiritual practices have been associated with positive health outcomes in many chronically ill adults.^43–46^ There should be a strategy to find integration of spiritual care in medical setting and this can start by assessing patients' preference of discussing religious and spiritual care during their clinical visits.

### Study Limitation

This study did not involve service providers whose information could have added different perspectives particularly on the home-based self-management of pain crisis and integration of psychosocial care in medical settings. Further researches should include their views and experiences. Few individuals with SCD were selected purposively, hence limit generalization of the findings.

However, findings from this study might provide an insight that can be used to improve comprehensive care given to individuals living with SCD.

## CONCLUSION

Individuals living with SCD experience several episodes of pain that affect their quality of life. Several approaches are used by individuals with SCD to self-manage the pain including taking rest, drinking plenty of water or using pain relieving medication. Care for patients with SCD should be comprehensive and should include proper management of pain, health education on home-based intervention for sickle cell pain crisis prevention, supportive services to deal with psychosocial implications of SCD and improve coping strategies to live with the illness.
